# Incidence, risk factors, clinical and microbiological characteristics of clostridium difficile associated diarrhea in spanish critically ill patients (procrid study)

**DOI:** 10.1186/2197-425X-3-S1-A116

**Published:** 2015-10-01

**Authors:** L Martin-Villen, A Gutierrez-Pizarraya, L Alcala-Hernandez, M Marin-Arriaza, B Balandin-Moreno, C Aragon-Gonzalez, J Ferreres-Franco, MA Chiveli-Monleon, MP Anguita-Alonso, E Bouza-Santiago, J Garnacho-Montero

**Affiliations:** Critical Care, Hospital Universitario Virgen del Rocio, Seville, Spain; Spanish Network Research in Infectious Diseases (REIPI), Seville, Spain; Microbiology, Hospital Universitario Gregorio Marañon, Madrid, Spain; Critical Care, Hospital Puerta de Hierro, Madrid, Spain; Critical Care, Hospital Carlos Haya, Malaga, Spain; Critical Care, Hospital Clinico Universitario, Valencia, Spain; Anesthesia and Resuscitation, Hospital Universitario La Fe, Valencia, Spain; Astellas Pharma S.A., Madrid, Spain

## Objectives

C. difficile (CD) is the first pathogen responsible of nosocomial diarrhea. Our aim was to study the epidemiology and factors associated with the development of CD infection (CDI) in patients admitted in the Critical Care Units (CCU) of our country.

## Materials

Multicenter, prospective, observational study from February 3^rd^ to April 3^rd^, 2014. We included all adult critically ill patients of 26 CCUs of Spain who had diarrhea^1^. All feces samples were sent to the reference laboratory and we considered CDI when cytotoxicity in cell culture or toxigenic culture were positive. The ribotypes were determined by PCR.

Incidence of CDI, clinical characteristics, possible risk factors and ribotypes were studied. For the bivariate analysis, the Chi-square test was used for qualitative variables and Student t-test or Mann Whitney U for quantitative. Multivariate analysis was performed using logistic regression to identify factors independently associated with the development of CDI.

## Results

In the study period, 7196 patients were admitted in the participating units, 190 (2.6%) had diarrhea and of them, 16 were positive for CD, representing a CDI incidence of 0.22%. 95´8% patients received antibiotics previously and only 2 patients (1.1%) had previous history of CDI. There was no difference in the severity mesured by APACHE II [17 (13-19) vs. 20 (16-25); p = 0.723] or the crude mortality (40 vs. 30.6%; p = 0.555) between patients with or without CDI.

CDI patients had a median age of 66 years, 43´8% were women and they had an income APACHE II median of 17 points. COPD (37.5%) was the most frequent comorbidity. CDI was a mild-moderate disease in the 64´3% of cases, 31´2% of the CDI patients had complications, 15.4% had recurrence and only one death was directely attributed to the CDI. The most frequently isolated ribotype was 078/126 (25%) and 027 were identified only in 2 cases (12.5%).

There were no differences in clinical presentation, previous use of ATB, use of inhibitors of proton pump, mechanical ventilation and parenteral nutrition among groups. Prevalence of chronic kidney disease (CKD) was significantly higher in infected patients (31.3% vs 7-1%; p 0.08) and by multivariate statistical analysis it was identified as the only factor independently associated with the development of CDI [OR 5,87 (1,24-27,83) 0,026].

## Conclusions

Despite using the clinical criteria of diarrhea^1^, the incidence of ICD in our population is very low.

We have identified several ribotype of CD including two cases of 027.

We only have indentified the chronic kidney disease as a risk factor independently associated with the development of CDI whereas the previous use of antibiotic seems not to directly influence the development of CDI.
